# Turnpike Catheter failure, causes and mechanisms: Insights from the MAUDE database

**DOI:** 10.1016/j.amsu.2022.103923

**Published:** 2022-06-05

**Authors:** Rashid Alhusain, John Dayco, Abdalaziz Awadelkarim, Talal Almas, Adnan Halboni, Ahmed K. Ahmed, Mohamed Elhussein, Mohamed Zghouzi, Waqas Ullah, Yasar Sattar, Mamas A. Mamas, Nasser Lakkis, M Chadi Alraies

**Affiliations:** a Wayne State University, Detroit Medical Center, Michigan, USA; b Royal College of Surgeons in Ireland, Dublin, Ireland; c Department of Radiology, Mayo Clinic, Jacksonville, FL, USA; d University of National Ribat, Khartoum, Sudan; e Thomas Jefferson University, Philadelphia, PA, USA; f West Virginia University, Morgantown, WV, USA; g Keele Cardiovascular Research Group, Centre for Prognosis Research, Institute for Primary Care and Health Sciences, Keele University, Stoke-on-Trent, UK; h Department of Cardiology, Royal Stoke University Hospital, Stoke-on-Trent, UK

**Keywords:** Turnpike, Microcatheter, Interventional cardiology

## Abstract

**Background:**

The Turnpike catheters (Teleflex, Wayne, PA, USA) is a microcatheter that was approved by the Food and Drug Administration in November 2014 to be used to access discrete regions of the coronary and peripheral vasculature.

**Methods:**

The Manufacturer and User Facility Device Experience (MAUDE) database was queried for reports of the Turnpike catheters from March 2015 through August 2021.

**Results:**

A total of 216 reports were found during the study period. After excluding duplicate reports (n = 21), our final cohort included 195 reports. The most common failure mode was catheter tip break or detachment (83%, *n* = 165) which was significantly associated with over-torquing (p-value = 0.025). The most common clinical consequence was the entrapment of the catheter (33%, *n* = 65), followed by vessel injury (7.8% n = 15) and vessel occlusion (3.6%, n = 7). Most patients had no consequences (47.0%, n = 93) or recovered (11%, n = 22). A total of 4 deaths were reported. 35.8% of reports (n = 69) specified the presence of severe calcification in the target vessel. Over torquing by interventionists was reported in 33.2% of events (n = 64).

**Conclusion:**

Despite clinical trials demonstrating the safety of the Turnpike catheters, complications can still occur. These data serve to inform operators about potentional risks and complications associated with the use of the device. Physicians should avoid over-torqueing which seems to be the most common mechanism for device complications.

## Highlights

• The Turnpike catheters (Teleflex, Wayne, PA, USA) is a microcatheter that was approved by the Food and Drug Administration in November 2014 to be used to access discrete regions of the coronary and peripheral vasculature.
• Despite clinical trials demonstrating the safety of the Turnpike catheters, complications can still occur. These data serve to inform operators about potential risks and complications associated with the use of the device.
• Physicians should avoid over-torqueing which seems to be the most common mechanism for device complications.

## 1 Introduction

**Fig. 1:**
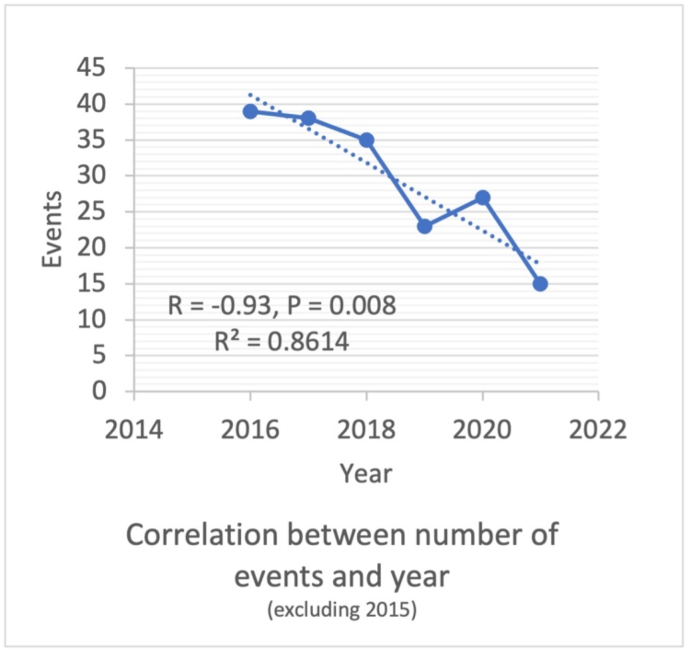
Dot-plot demonstrates the relationship between the number of reported events and year of reporting excluding events in 2015.

Outcomes of chronic total occlusion (CTO) percutaneous coronary intervention (PCI) have improved because of advancements in equipment and techniques [1,2]. Coronary microcatheter (MCs) are often used to access discrete regions of the coronary vasculature during CTO and complex PCIs and subsequently deliver diagnostic and therapeutic interventions [1,3]. These catheters are usually structured in a multilayer design to enhance guidewire navigation, exchanges and enhance their penetration force during coronary intervention in tortuous, CTO, or calcific lesions. Coronary MCs are classified as high profile, low profile, angulated, dual lumen, and plaque-modifying [3,4] (see Fig. 1).

Turnpike catheter (Teleflex Incorporated, Wayne, PA, USA) is a coronary microcatheter that was approved by the Food and Drug Administration (FDA) in November 2014 [5]. The current Turnpike Catheter portfolio consists of the Turnpike catheter (standard version), the Turnpike Spiral Catheter (TSC), the Turnpike Gold Catheter (TGC), and the Turnpike LP Catheter (TLC). Each catheter contains a different design element that has been designed to address different clinical challenges during endovascular procedures. They can be grouped according to their recommended approach during CTO procedures as antegrade (TSC, TGC), retrograde (TLC), or both (Turnpike standard) [[6], [7], [8]].

Despite extensive clinical use, real-world data on the most common mode of failures and complications associated with the use of Turnpike catheters is unknown. We investigated the Manufacturer and User Facility Device Experience (MAUDE) database for reports on Turnpike catheters to better understand their complications and failure modes.

## 2 Methods

### 2.1 Data source

The FDA created the MAUDE online database enlisting adverse events caused by approved medical devices. The MAUDE database contains reports submitted to the FDA by mandatory reporters (manufacturers, importers, and device user facilities) and voluntary reporters such as health care professionals, patients, and consumers [9]. The MAUDE database is publicly available and de-identified. Therefore, no institutional review board approval was required for this study. We queried the database from March 2015 through August 2021 using the keyword “Turnpike.” The database was last accessed on October 18th, 2021, by two independent reviewers.

### 2.2 Outcomes and statistical analysis

The primary outcome of this study was the mechanisms of failure of the Turnpike catheters. Secondary outcomes included clinical consequences of device failure. The MAUDE database is unable to capture all the cases of Turnpike utilization in the U.S. Hence it cannot predict the actual incidence rate of failures or complications. Categorical variables were described as numbers, and all statistical calculations were performed with IBM SPSS Statistics for Windows, Version 27.0. Armonk, NY: IBM Corp [10].

## 3 Results

A total of 216 reports were found during the study period. After excluding duplicate reports (n = 21), our final cohort included 195 reports. The most common Turnpike catheter associated with adverse events was the Turnpike Spiral (39.1%, n = 76), followed by Turnpike LP (31.1%, n = 62), Turnpike Smooth (9.3%, n = 18), and lastly, Turnpike Gold (1%, n = 2). 36 reports (18%) were unidentified Turnpike catheters. The nature of procedures and clinical course were not described in all reports.

The most common failure mode was catheter tip detachment or break (83%, *n* = 165), which was significantly associated with over-torquing (p-value = 0.025). The most common clinical consequence was the entrapment of the catheter (33%, *n* = 65), followed by vessel injury (7.8% n = 15) and vessel occlusion (3.6%, n = 7). Most patients had no consequences (47.0%, n = 93) or recovered (11%, n = 22). 4 deaths were reported; one was due to acute in-stent thrombosis, another was due to aortic dissection, which was presumed to be non-related to the catheter. The third death occurred after CABG due to an unsuccessful CTO-PCI where the Turnpike was utilized, and the last patient suffered perforation of the target vessel that resulted in cardiac tamponade. We noticed a statistically significant decrease in events per year after excluding events in 2015 (R = −0.93, P-value = 0.008). Right coronary artery (RCA) was the most common target vessel of intervention implicated in events (16.1%; n = 31) followed by left anterior descending artery (LAD) (14.0%; n = 27), Left circumflex (LCX) (5.2%; n = 10), Left main coronary artery (LMCA) (1.0%; n = 2) and others including peripheral (8.3%; n = 16). Most reports (55.4%, n = 107) did not specify the target vessel of intervention. 35.8% of reports (n = 69) specified the presence of severe calcifications in the target vessel. Over torquing by interventionists was reported in 33.2% of events (n = 64) (Table 1).

**Table 1:** Reports of the Turnpike catheters failure in MAUDE registry.

Total number of events	194
Year of event	
2015, n(%)	14 (7.2%)
2016, n(%)	39 (20.0%)
2017, n(%)	38 (19.5%)
2018, n(%)	35 (17.9%)
2019, n(%)	23 (11.8%)
2020, n(%)	27 (13.8%)
2021, n(%)	15 (7.7%)
Unidentified, n(%)	2 (1%)
Turnpike VCD type	
Turnpike Spiral, n(%)	76 (39.4%)
Turnpike Smooth, n(%)	18 (9.3%)
Turnpike LP, n(%)	62 (31.1%)
Turnpike Gold, n(%)	2 (1.0%)
Unidentified, n(%)	36 (18.1%)
Target vessel	
RCA, n(%)	31 (16.1%)
LAD, n(%)	27 (14.0%)
LCX, n(%)	10 (5.2%)
LMCA, n(%)	2 (1.0%)
Other, n(%)	16 (8.3%)
Insufficient information, n(%)	107 (55.4%)
Severely calcified vessel, n(%)	69 (35.8%)
Over-torquing, n(%)	64 (33.2%)
Failure method	
Detachment, or break, n(%)	164 (85%)
Entrapment, n(%)	7 (3.5%)
Failed engagement, n(%)	9 (4.7%)
Insufficient information, n(%)	1 (0.5%)
None, n(%)	12 (6.2%)
Clinical consequence	
Major bleeding (Hypotension), n(%)	0 (0%)
Vessel occlusion, n(%)	7 (3.6%)
Vessel injury, n(%)	15 (7.8%)
Hematoma, n(%)	1 (0.5%)
Retroperitoneal hematoma, n(%)	0 (0%)
Catheter Entrapment, n(%)	65 (33.2%)
Insufficient information, n(%)	30 (15.5%)
None	76 (39.4%)
Patient outcome	
Death, n(%)	4 (2.0%)
No consequences, n(%)	93 (47.0%)
Recovered, n(%)	22 (11.1%)
Insufficient information, n(%)	75 (37.9%)

MAUDE: Manufacturer and User Facility Device Experience, RCA: Right coronary artery, LAD: Left anterior descending artery, LCX: Left circumflex artery, LMCA: Left main coronary artery.

## 4 Discussion

Since its FDA approval in November 2014(11), Turnpike catheters have been widely utilized in the vascular access of CTOs or complex PCIs. In a study of 2968 CTO patients between January 2016 to January 2019, the turnpike spiral was the most commonly utilized microcatheter (18%), followed by the Turnpike Standard (16%) [2]. This explain our observation that the Turnpike Spiral was associated with the most complications (39%, n = 77). Despite the extensive utilization of Turnpike catheters, no study has been performed to outline and report its failure modes and complications.

Our analysis demonstrates a decrease in the reported incidence rate observed between January 2016 to August 2021. This may be attributed to an increasing physician awareness and familiarity with the Turnpike catheter, which was approved in November of 2014 [11]. For instance, the most common complication of catheter tip detachment is highly attributed to the operator is over-torquing the catheter through difficult advancements, such as a highly calcified or torturous lesion [12]. Events in 2015 were excluded from this analysis given the low number of reported events, likely due to the device's recent approval. Our study found that over-torquing has been reported in third of all the reported complications. The manufacturer has repeatedly warned against rotating the catheter more than two full rotations (520°) in either direction if the distal tip is not rotating or advancing [13]. Catheter tip detachments are not only observed in Turnpike catheters but also in most of the currently utilized microcatheters (MC) [12]. The likely explanation is that as interventionalists become more familiar with the technical operations of MCs, over-torquing is increasingly being avoided.

There is also a significant association between the target vessel and complications. In our study, we found that complications were reported the most in interventions involving the RCA and the LAD artery. Most of the reported complications occurred in the RCA, followed by the LAD. The high rate of complications observed in the RCA may be due to anatomical considerations, where tortuosity and angulations are often observed at the beginning and later segments. The RCA's origin particularly in anomalous takeoffs may mean difficulty in manipulating the catheter [14].

MCs, such as Turnpike catheters, have enabled us to access calcified vessels such as a CTO and tortuous vessels. However, it is still worth noting that such microcatheters should be cautioned in heavily calcified lesions due to the higher likelihood of catheter tip detachment or entrapment [15]. The MAUDE database does not provide information on the degree of calcification, which can be classified as light, medium, or substantial [7]. At this time, the guidelines are unclear regarding the utilization of MC and the degree of calcifications. The decision to avoid MC intervention with the degree of calcification or stenosis is a current gap in the literature and would need to be addressed.

A theoretical device limitation of Turnpike catheters is the likelihood of catheter tip fracture with misuse. This is inherent to the small structure of microcatheters, particularly in the tip, which allows it to glide through severe stenosis and tortuous vessels. This highlights the importance of careful utilization of the catheters, especially when passing through a high degree of stenosis. If a catheter tip fracture was to occur, some reported leaving the fractured tip in the vessel, covered by a stent [1,15]. Otherwise, the tips could also be retrieved with various techniques, such as a guidewire [16].

Amongst the reported complications, 4 total deaths (2%) were observed. However, it is essential to note that this mortality rate was observed in the immediate postoperative stage of the catheterization. There is no sufficient data on the mortality rate on long-term follow-up or the risk of stent thrombosis associated with retained fractured tips.

## 5 Limitations

Our study is a retrospective analysis from the MAUDE database. The major limitation is the selection bias based on the optional reporting by healthcare professionals. Given the voluntary nature of reporting, there is potential for the significant underreporting of these adverse events. Additionally, the nature of the database limits the accuracy of the correlation between device failure and clinical adverse events. Finally, MAUDE data alone cannot be used to evaluate a change in event rates over time or compare event rates between devices. The overall incidence of MC failure could not be evaluated as the database lacks data on the overall utilization of MCs. The number of reports cannot be interpreted or used in isolation to reach conclusions about the existence, severity, or frequency of problems associated with devices.

Nonetheless, despite these limitations, our study was able to include a sample of 195 reported complications, spanning 7 years. This may provide valuable insights on the most common failure modes and complications, and be of particular value in guiding interventionalists who may be utilizing the Turnpike catheters. Over-torquing appears to be the most common mechanism implicated in failure of the device and complications.

## 6 Conclusion

Despite clinical trials demonstrating the safety of the Turnpike catheters, complications can still occur. These data serve to inform operators about protentional risks and complications associated with the use of the device. Physicians should be well-trained to use Turnpike catheters and avoid over-torquing, leading to catheter break and detachment.

## Sources of funding for your research

NA.

## Ethical approval

NA.

## Funding

None.

## Author contribution

RA, JD, AA,TA, AH: conceived the idea, designed the study, and drafted the manuscript.AKA, ME, MZ, WU: conducted literature search and created the illustrations.YS, MAM: revised the manuscript critically and refined the illustrations.TA, NL, MCA: revised the final version of the manuscript critically and gave the final approval.

## Conflicts of interest

NA.

## Consent

Written and informed consent was obtained and is available to the editor in chief upon request.

## Registration of research studies

1. Name of the registry: NA
2. Unique Identifying number or registration ID: NA
3. Hyperlink to your specific registration (must be publicly accessible and will be checked): NA

## Guarantor

Talal AlmasRCSI University of Medicine and Health Sciences123 St. Stephen's GreenDublin 2, IrelandTalalalmas.almas@gmail.com.

## Authors’ contribution

RA, JD, AA,TA, AH: conceived the idea, designed the study, and drafted the manuscript.AKA, ME, MZ, WU: conducted literature search and created the illustrations.YS, MAM: revised the manuscript critically and refined the illustrations.TA, NL, MCA: revised the final version of the manuscript critically and gave the final approval.

## Declaration of competing interest

None of the authors have any conflict of interest.
